# A Rare Manifestation of Extrapulmonary Tuberculosis: Tenosynovitis of Wrist and Multiple Fingers

**DOI:** 10.7759/cureus.84025

**Published:** 2025-05-13

**Authors:** Taha El Aissaoui, Achraf Tebbaa El Hassali, Adnane Lachkar, Hicham Yacoubi, Najib Abdeljaouad

**Affiliations:** 1 Department of Traumatology and Orthopaedics, Mohammed VI University Hospital, Faculty of Medicine and Pharmacy, Mohammed First University, Oujda, MAR

**Keywords:** caseous necrosis, rice bodies, surgical synovectomy, tenosynovitis of wrist, tuberculosis

## Abstract

Tuberculous tenosynovitis is a rare extrapulmonary manifestation of tuberculosis and often presents with nonspecific symptoms that mimic more common inflammatory or degenerative conditions. We report a unique case from Eastern Morocco involving a 39-year-old male with a two-year history of progressive swelling and limited flexion of the wrist and the second, third, and fifth fingers. Imaging revealed tenosynovitis of the flexor tendons, and histopathological examination confirmed tuberculosis through the presence of granulomatous inflammation with caseous necrosis. The patient underwent a surgical synovectomy followed by a six-month course of anti-tuberculosis therapy, resulting in significant clinical improvement and restored joint mobility. This case underscores the importance of considering tuberculosis in the differential diagnosis of chronic tenosynovitis, particularly in endemic regions. It highlights the role of early histopathological confirmation and combined medical-surgical management in achieving favorable outcomes.

## Introduction

Tuberculosis (TB) remains a major global public health issue despite significant advancements in diagnostic methods and therapeutic strategies. Tuberculous tenosynovitis is an uncommon manifestation of extrapulmonary tuberculosis, accounting for approximately 5% of osteoarticular tuberculosis cases, and most frequently involves the flexor tendons of the wrist and hand [1,2]. Musculoskeletal tuberculosis represents 1% to 3% of all TB cases [3]. This condition typically has an insidious onset characterized by chronic swelling, pain, and restricted joint mobility. These nonspecific symptoms often lead to delayed diagnosis or misdiagnosis as more common inflammatory or degenerative joint disorders [4]. The diagnosis is most reliably confirmed through histopathological examination [5]. The cornerstone of treatment involves a multidrug antituberculosis regimen, typically including isoniazid, rifampicin, pyrazinamide, and ethambutol, administered throughout six to nine months [6]. Surgical intervention is indicated in cases where medical therapy proves ineffective, particularly when there is a restricted range of motion or evidence of neurovascular compression [7,8]. The presence of rice bodies necessitates thorough debridement and complete excision of the involved bursae to minimize the risk of recurrence [9,10].

We report a rare case of tuberculous tenosynovitis involving the flexor tendons of the wrist and the second, third, and fifth fingers in a patient from Eastern Morocco. This represents the first documented case in this geographical region, underscoring the importance of heightened clinical awareness in endemic areas.

## Case presentation

**Figure 1:**
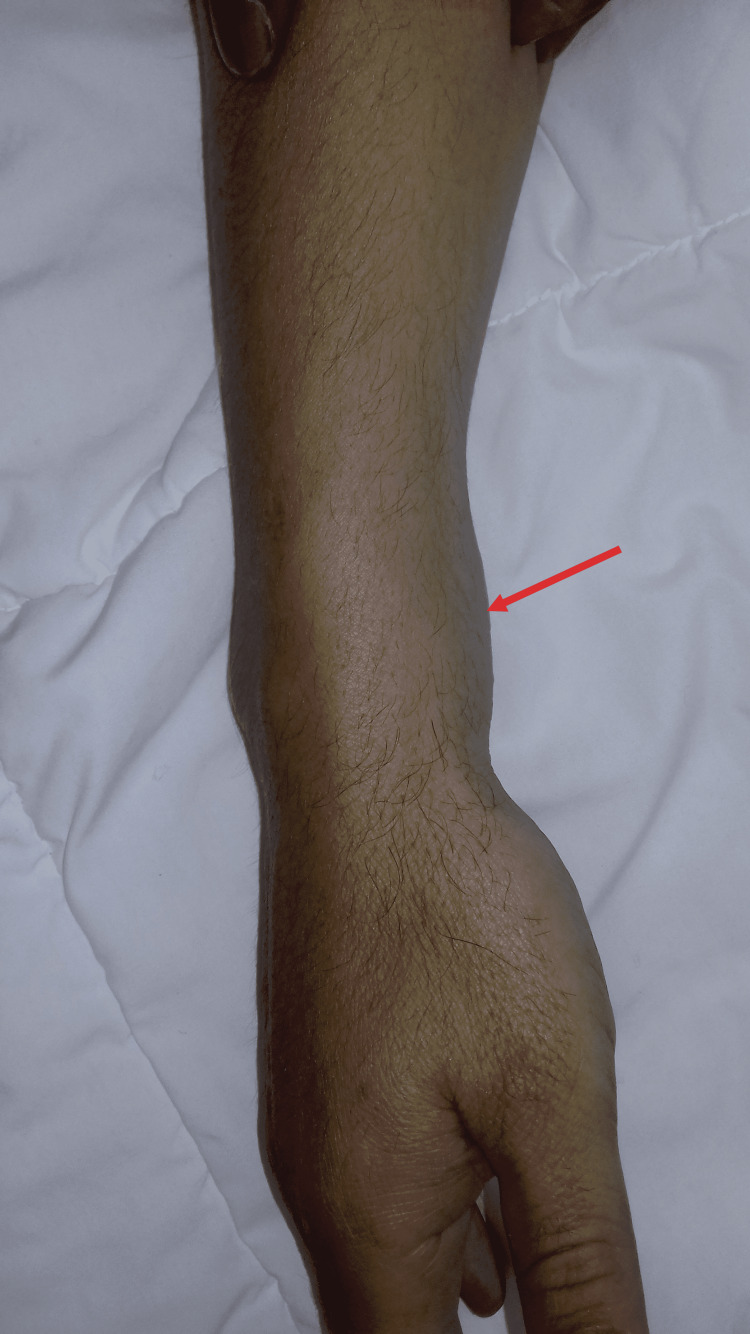
Clinical image showing swelling of the anterior aspect of the right wrist

**Figure 2:**
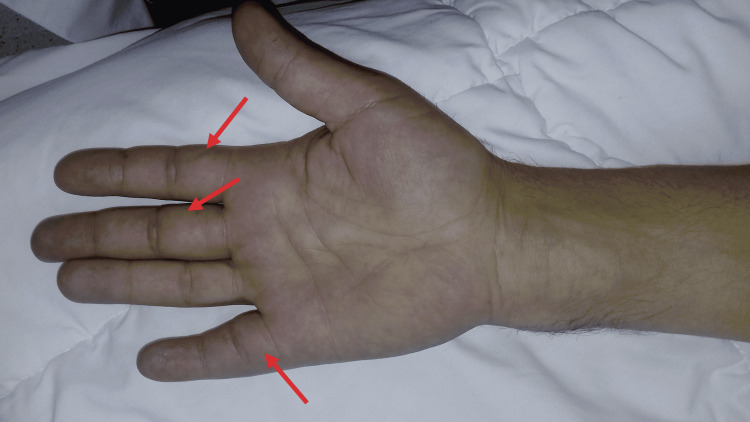
Clinical image showing swelling of the anterior aspect of the second, third, and fifth right fingers.

We report the case of a 39-year-old male with no notable medical history who presented with progressive swelling of the anterior compartment of the right wrist and the second, third, and fifth right fingers. This condition had evolved over two years and was associated with increasing wrist and finger flexion limitations. The patient did not report any cutaneous inflammatory signs, fever, or weight loss. Physical examination revealed a mobile, painless, soft mass located over the volar aspect of the right wrist and the affected fingers (Figures 1, 2). There was a noticeable limitation in the range of motion at the proximal and distal interphalangeal joints of the second, third, and fifth right fingers. Vascular and neurological assessments were normal.

**Figure 3:**
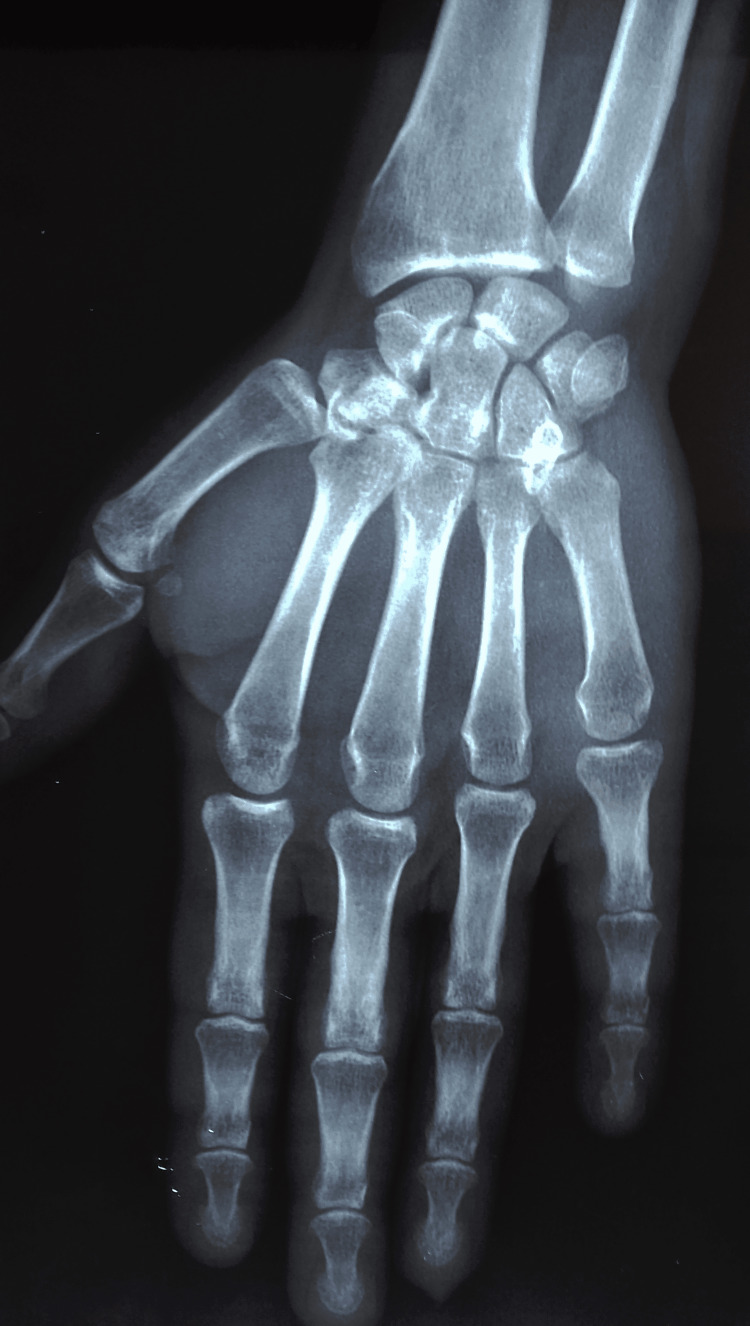
Radiograph showing anteroposterior view of the right wrist and hand

**Figure 4:**
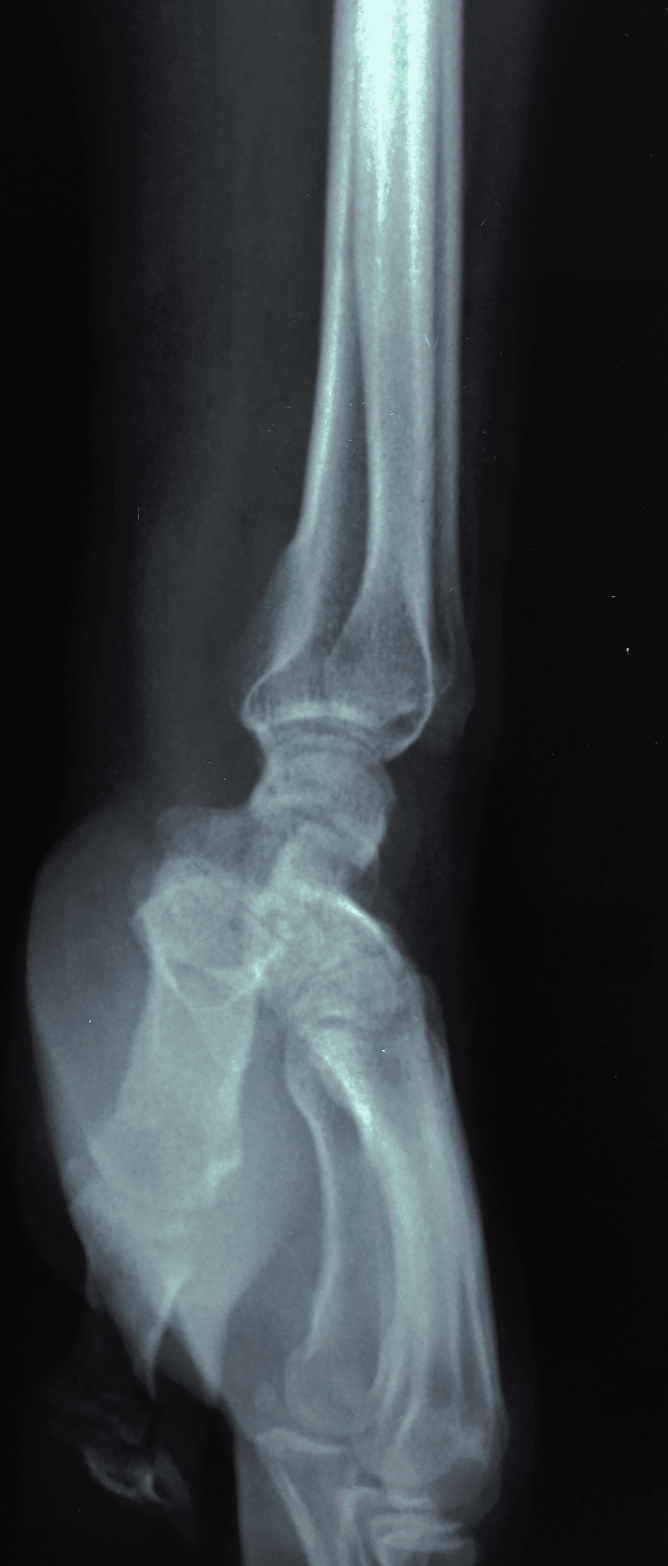
Radiograph showing lateral view of the right wrist and hand

**Figure 5:**
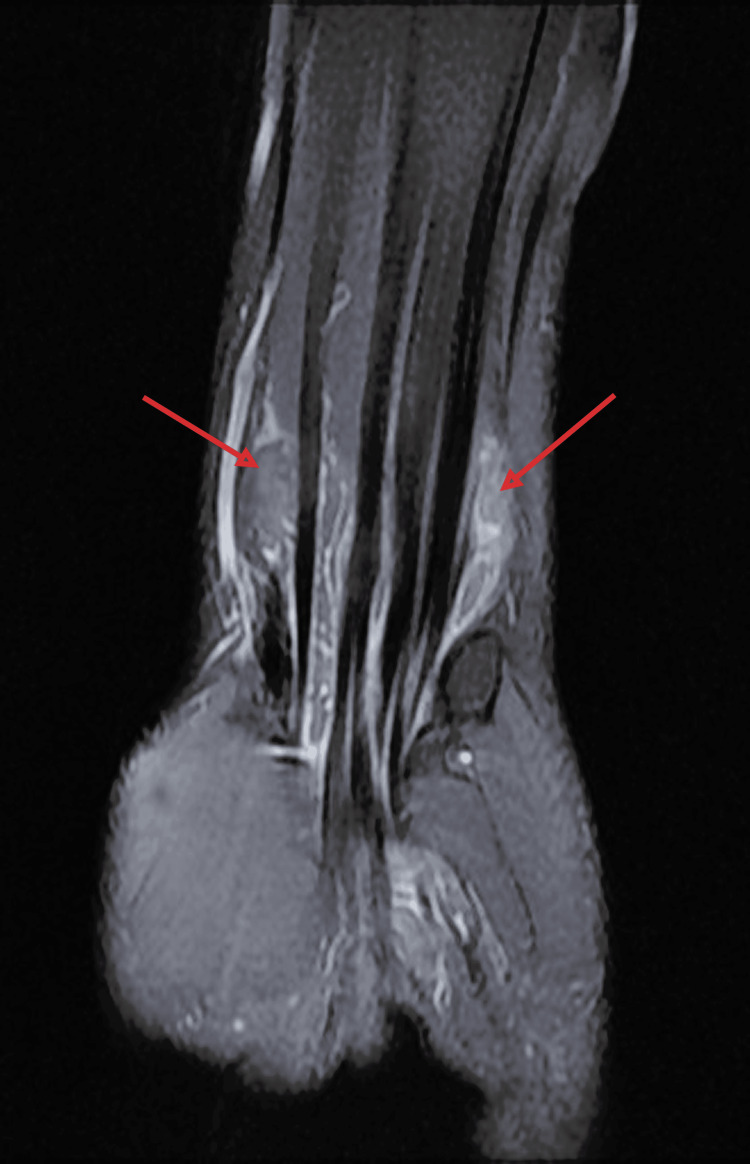
Proton density fat-saturated coronal MRI of the wrist The image is showing fluid collections along flexor tendon sheaths, suggestive of tenosynovitis.

**Figure 6:**
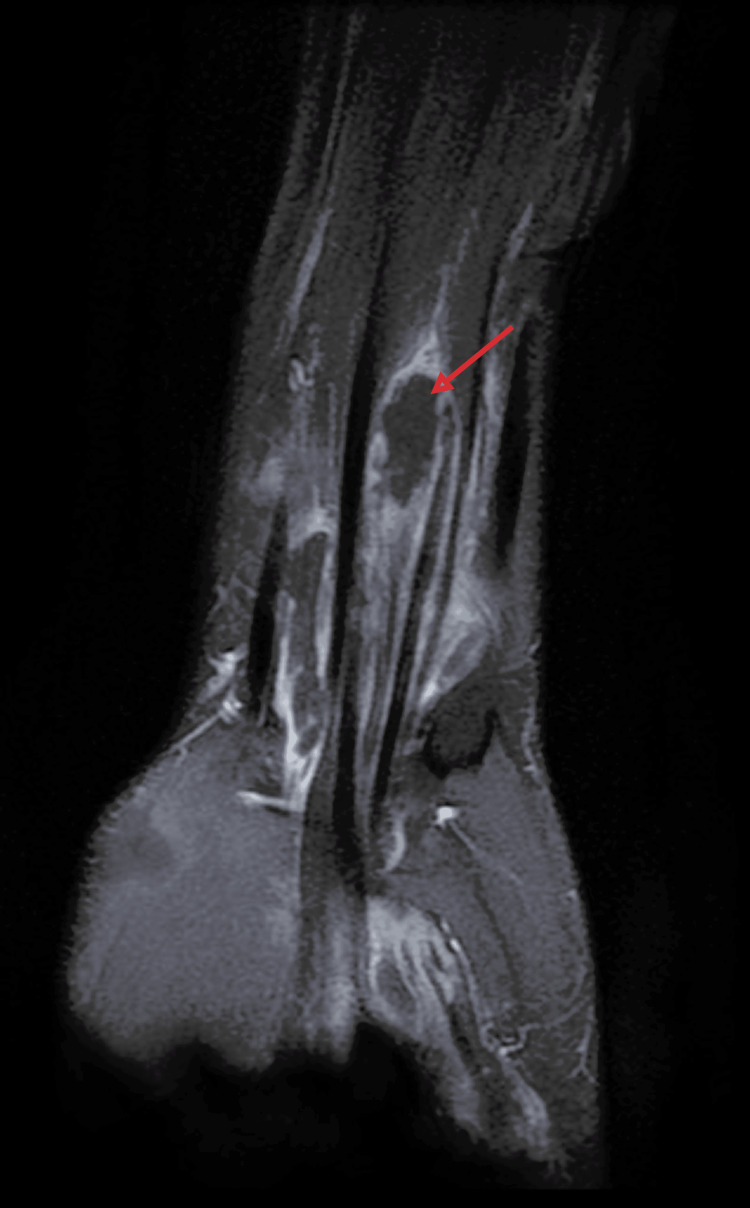
T1 fat-saturated coronal MRI of the wrist The image is showing fluid collection along the flexor tendon sheath, suggestive of tenosynovitis.

**Figure 7:**
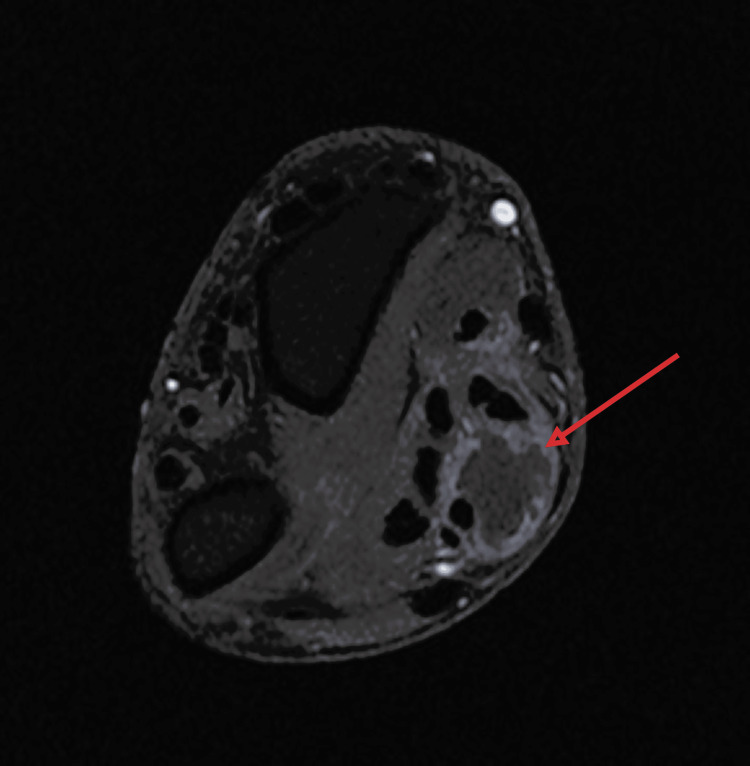
T1 fat-saturated axial MRI of the wrist The image is showing a well-defined fluid collection along the flexor tendon sheaths, suggestive of tenosynovitis.

Biological investigations revealed a white blood cell count of 9,000/mm³ and an elevated C-reactive protein (CRP) level of 40 mg/L; polymerase chain reaction (PCR) testing for *Mycobacterium tuberculosis* was positive. Standard anteroposterior and lateral radiographs of the wrist and hand showed no abnormalities (Figures 3, 4). Ultrasound and magnetic resonance imaging (MRI) demonstrated tenosynovitis of the flexor tendons in the wrist and hand (Figures 5-7)

**Figure 8:**
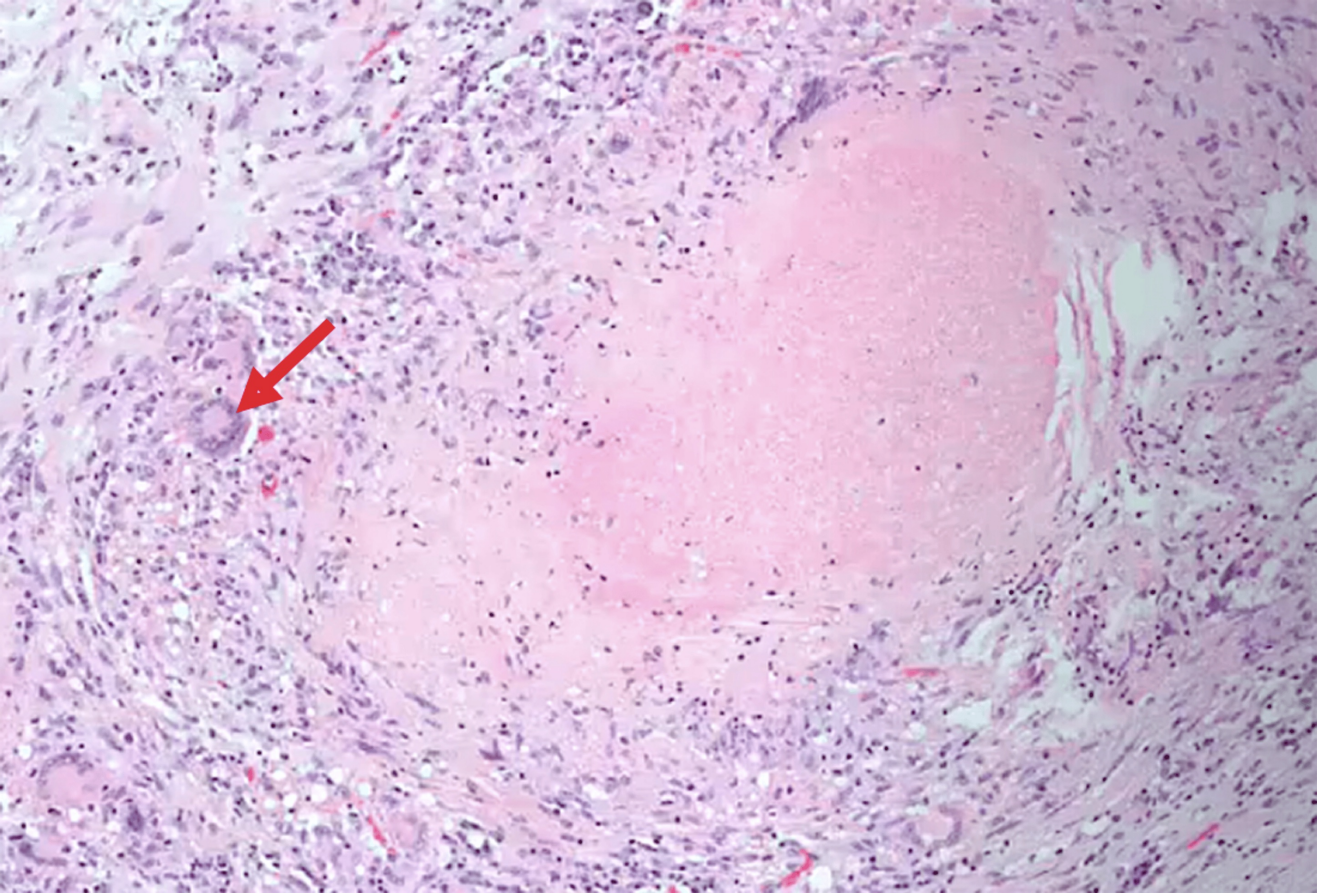
Histopathology study Histopathological image showing granulomas with epithelioid cells and multinucleated giant cells, arranged nodularly and exhibiting extensive central caseous necrosis.

**Figure 9:**
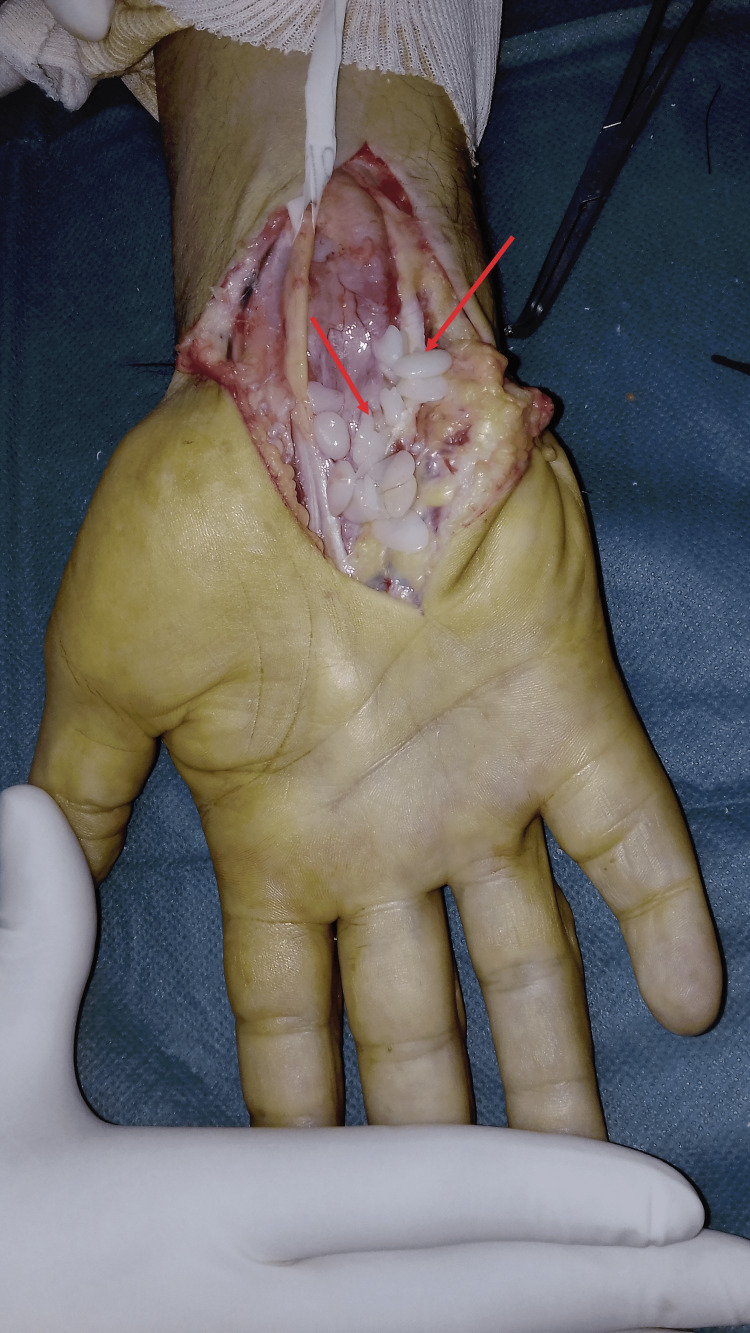
Peroperative image showing rice bodies in the anterior aspect of the wrist

**Figure 10:**
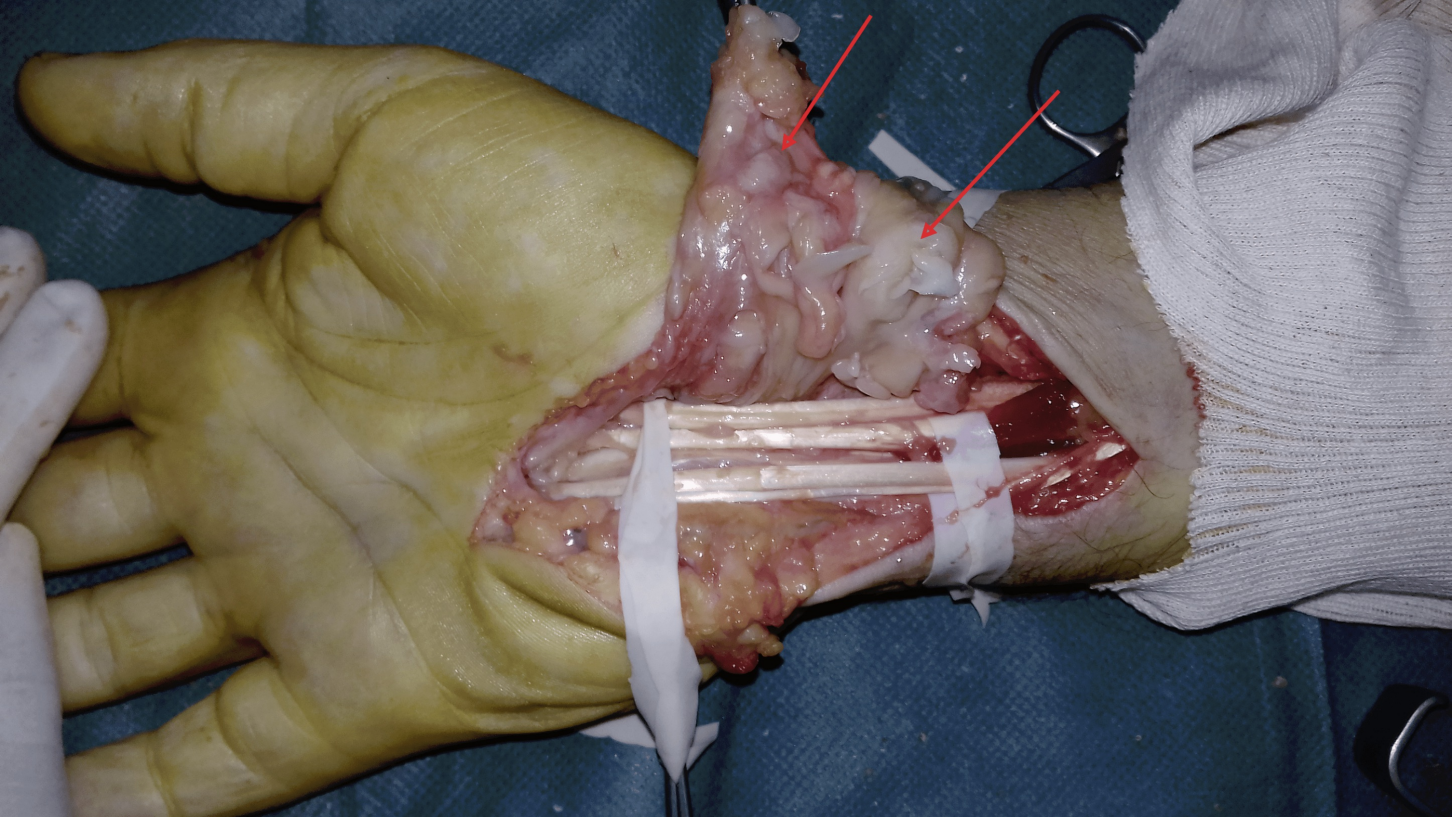
Peroperative image showing flexor tendons exposed after synovectomy

**Figure 11:**
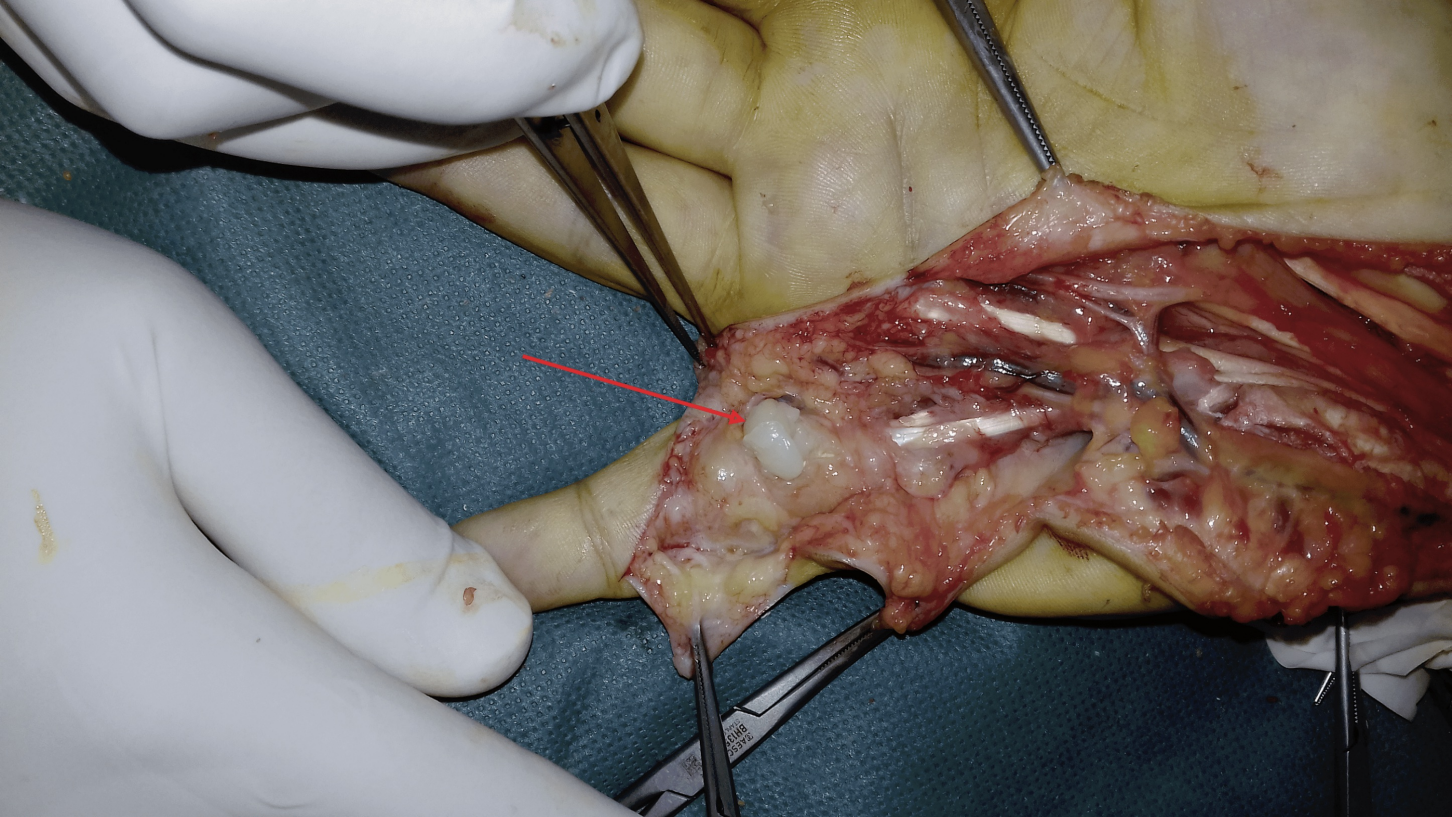
Peroperative image showing rice bodies in the anterior aspect of the right fifth finger

**Figure 12:**
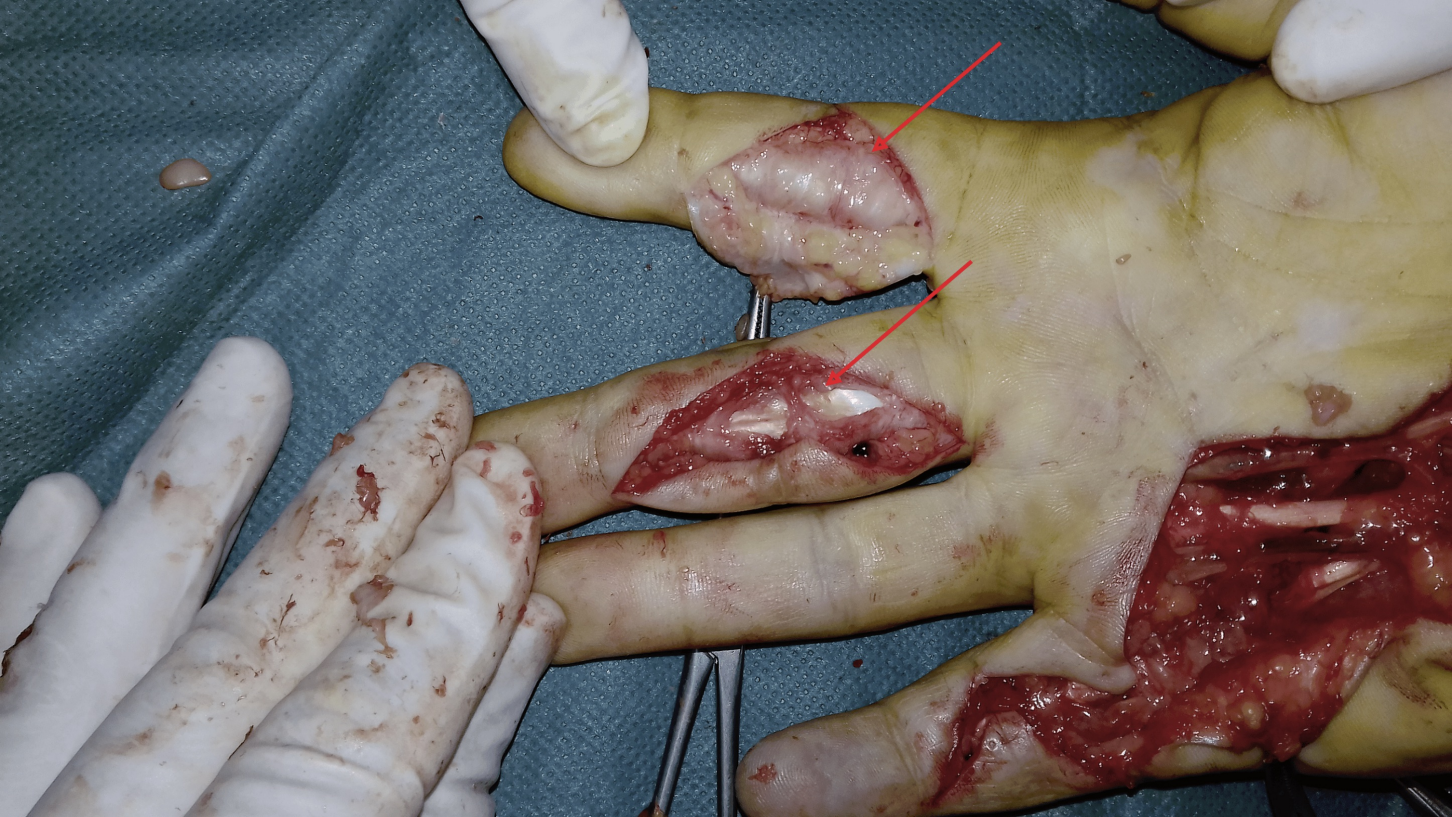
Surgical view of anterior approach for synovectomy of the second and third fingers

In light of the clinical and radiological findings, a biopsy was performed. Mycobacterial culture for *Mycobacterium tuberculosis* was positive, and histopathological analysis revealed multiple granulomas with epithelioid cells and multinucleated giant cells, arranged in a nodular pattern and displaying extensive caseous necrosis at the center, confirming the diagnosis of tuberculous tenosynovitis (Figure 8). Surgical management was indicated due to the limitation in the flexion range of motion of the wrist and fingers and the histopathological findings. A synovectomy of the flexor tendons at the wrist was performed via an anterior approach, revealing characteristic "rice grain" bodies (Figures 9, 10). This was followed by synovial resection of the flexor tendons of the second, third, and fifth fingers (Figures 11, 12).

After consultation with the pulmonologist and in light of the absence of other tuberculosis localizations, the patient was initiated on a six-month course of anti-tuberculosis therapy and underwent physical rehabilitation to restore joint mobility and muscle strength. Follow-up revealed a favorable outcome, with significant articular range of motion recovery, pain reduction, and no evidence of disease recurrence.

## Discussion

The introduction of antitubercular therapy in the 1950s significantly reduced the global incidence of tuberculosis. However, the emergence of immunosuppressive conditions and increasing resistance to anti-tuberculosis medications have counterbalanced these advances, sustaining the disease burden in many regions. The musculoskeletal system represents the third most common site of extrapulmonary tuberculosis following pulmonary and lymphatic involvement and accounts for approximately 10-20% of extrapulmonary tuberculosis cases [10].

Tuberculous tenosynovitis of the wrist and fingers is a rare manifestation of musculoskeletal tuberculosis and presents considerable diagnostic challenges. The infection typically results from hematogenous dissemination, though lymphatic spread or direct inoculation may also occur [11]. Without timely diagnosis and treatment, tuberculous tenosynovitis can lead to extensive damage to tendons and joints [12,13].

The condition presents with chronic pain and swelling, which closely mimic other common pathologies, such as rheumatoid arthritis, ganglion cysts, or nonspecific tenosynovitis [14]. The flexor tendons are more frequently affected than the extensor tendons, a predisposition attributed to their anatomical course and synovial sheath complexity [15]. Classic clinical presentations of tuberculous tenosynovitis include: a compound palmar ganglion commonly involving the flexor sheath of the little finger and extending into the ulnar bursa; the sausage digit usually involving a single finger (typically the index, middle, or ring), manifests as fusiform swelling; and finally Carpal tunnel syndrome [7].

Laboratory findings, including white blood cell count, erythrocyte sedimentation rate (ESR), and C-reactive protein (CRP), are nonspecific and do not exclude the diagnosis [6]. Similarly, conventional radiographs are often normal in early disease and may fail to detect bony involvement [16]. Ultrasound is a valuable, cost-effective imaging tool for the initial evaluation of tenosynovitis, allowing real-time assessment of tendon sheath thickening, fluid collections, and synovial proliferation. Its accessibility and non-invasive nature make it especially useful in resource-limited settings.

Magnetic resonance imaging (MRI) is more sensitive. It may reveal synovial hypertrophy, effusion, and the presence of rice bodies, though these are not pathognomonic, as they may also occur in rheumatoid arthritis or other chronic synovitides [17]. Definitive diagnosis relies on histopathological examination demonstrating granulomatous inflammation with caseating necrosis and Langhans-type giant cells. Confirmation may be supported by positive culture or PCR for *Mycobacterium tuberculosis* [5].

Treatment of tuberculous tenosynovitis is primarily medical. According to the 2019 World Health Organization guidelines, standard anti-tuberculosis therapy includes an intensive phase of two months with rifampicin, isoniazid, ethambutol, and pyrazinamide, followed by a four-month continuation phase with rifampicin and isoniazid [18]. Surgical intervention is reserved for cases complicated by neurovascular compression, risk of tendon rupture, bone involvement, or for debridement and correction of residual deformities [18]. No prior cases have been reported from Eastern Morocco. This case, therefore, contributes a unique geographic perspective, reinforcing the need to consider tuberculosis in the differential diagnosis of chronic tenosynovitis, especially in endemic regions.

## Conclusions

Tuberculous tenosynovitis of the wrist and fingers remains an infrequent but important differential diagnosis in patients with chronic hand and wrist swelling, particularly in endemic regions. This case from Eastern Morocco highlights the insidious nature and diagnostic complexity of the condition, often mimicking more common inflammatory or degenerative pathologies. Early histopathological confirmation and appropriate initiation of anti-tuberculosis therapy, complemented by surgical intervention when necessary, are critical for preventing functional impairment. Greater clinical vigilance and reporting of such cases are essential to improving recognition and outcomes in underrepresented geographical areas.
